# Dietary Mannoheptulose Does Not Significantly Alter Daily Energy Expenditure in Adult Labrador Retrievers

**DOI:** 10.1371/journal.pone.0143324

**Published:** 2015-12-11

**Authors:** Leslie L. McKnight, Jared Root-McCaig, David Wright, Gary M. Davenport, James France, Anna Kate Shoveller

**Affiliations:** 1 Centre for Nutrition Modelling, Department of Animal Biosciences, University of Guelph, Guelph, Ontario, N1G 2W1, Canada; 2 Department of Human Health and Nutritional Sciences, University of Guelph, Guelph, Ontario, N1G 2W1, Canada; 3 Procter and Gamble Pet Care, 6571 State Route 503 North, Lewisburg, Ohio, 45338, United States of America; Oklahoma State University, UNITED STATES

## Abstract

Mannoheptulose (MH), a sugar found in avocados that inhibits glycolysis *in vitro*, has been preliminarily investigated as a novel food ingredient for dogs. This study aimed to determine the effects of dietary MH, delivered as an extract of un-ripened avocado, on energy expenditure (EE) in healthy adult Labrador Retriever dogs (total of 12 dogs, 26.99 ± 0.634 kg, 4.9 ± 0.2 y). The study was a double-blind, cross-over with each dog receiving both dietary treatments, control (CON) and MH (400 mg/kg of diet; 6 mg/kg BW), in random order. Resting and post-prandial (10 h) EE and respiratory quotient (RQ) were determined by indirect calorimetry (d 42). The following day, body composition was assessed using dual X-ray absorptiometry. Continuous activity monitoring was conducted using an Atical® accelerometer (d 43–47). A vastus lateralis muscle biopsy was obtained prior to the morning meal (d 49) and 4 h after consumption of their meal (d 56) to determine the protein content and phosphorylation of 5' adenosine monophosphate-activated protein kinase (AMPK). Diet did not affect body weight, resting EE or skeletal muscle AMPK phosphorylation. Dogs fed MH had significantly lower post-prandial RQ (p = 0.02) and ratio of fat to lean body mass (p = 0.02). Physical activity during light time periods (but not dark) was lower in dogs fed MH (p < 0.05) during weekends, but not on weekdays. These results suggest that MH affects energy balance of adult dogs, but that these effects are not dose dependent and not due to physical activity.

## Introduction

The average lifespan of dogs has been increasing; it has been estimated that more than 50% of dogs in the United States are over the age of six [1]. As in humans, ageing in canines is thought to be accelerated by aspects such as genetics, disease, and/or harmful environmental and lifestyle factors. Nutrition management of dogs, throughout the life cycle, should seek to delay (or prevent) the physiological and metabolic changes associated with aging.

Energy restriction (ER), without malnutrition, has been shown to increase median lifespan in yeast, flies, nematodes, rodents [2] and dogs [3]. Studies in humans and non-human primates have not found ER to increase lifespan, but have shown that ER provides protection from certain age related diseases [4,5]. Similarly, Labrador Retrievers subjected to 25% ER had and increased median lifespan (2 y), significantly lower triglycerides, cholesterol and superior glucose tolerance than control animals (fed to weight maintenance) [3]. Furthermore, ER dogs had significantly lower ratio of body fat to lean mass than dogs that were not restricted. Despite these benefits, ER has limited clinical success, presumably due to the significant behavioural changes it elicits in pets, including begging, aggression and scavenging. Such behaviours are often perceived as negative by the owner, thereby straining the owner-animal bond and presumably resulting in poor owner compliance with ER strategies [6].

Food ingredients enriched with compounds thought to favourably alter metabolism or extracts of these compound may provide an alternative to ER. In humans, several plant-derived polyphenols are currently under investigation, including, but not limited to, resveratrol and epigallocatechin gallate [7]. How these compounds exert their beneficial effects is not fully understood. However, many of these compounds are believed to work, in part, by activating the key energy sensing protein 5' adenosine monophosphate-activated protein kinase (AMPK) [7]. Once activated, AMPK promotes ATP-generating pathways and reduces anabolic processes, creating a direct link between cellular energy status and whole body energy expenditure (EE).

Mannoheptulose (MH), a seven-carbon sugar found in avocados, has been preliminarily investigated as a novel food ingredient for dogs. Early research demonstrated that MH competitively inhibits hexokinases [8,9]. Studies administering oral MH to dogs are limited and the results have been inconsistent. Labrador Retrievers fed a gelatin capsule containing 2, 10, and 20 mg/kg BW MH had lower fasting serum insulin than dogs fed a placebo capsule or a capsule containing 1 mg/kg BW MH [10]. In contrast, no changes in serum insulin or glucose or post-prandial energy expenditure (EE) were noted in Labrador Retrievers receiving a dietary dose of 200 mg MH/kg diet [11]. No changes in serum glucose or insulin concentrations were noted in Beagles given an oral cocoa butter based supplement containing 8 mg/kg BW MH. However, a transient increase in post-prandial energy expenditure was observed [12]. The mechanism by which MH would impact whole body energy expenditure has not been investigated. As glucose deprivation has been shown to activate AMPK [13], it is plausible that MH-induced inhibition of glycolysis would decrease ATP production thereby activating AMPK and in turn decreasing diet induced thermogenesis and increasing lipid oxidation and glucose uptake. However, this hypothesis has not been tested.

The primary objective of this study was to examine the effects of dietary MH (400 mg MH/kg diet; 6 mg/kg BW) on EE in adult Labrador Retrievers. In addition, we examined the effects of MH on skeletal muscle AMPKα protein content and phosphorylation. We hypothesized MH induced inhibition of hexokinases would limit glucose as a substrate of energy production and consequently increase fat utilization. Furthermore, MH was expected to decrease post-prandial EE and this decrease would be associated with increases in the activation of AMPK in skeletal muscle.

## Methods and Materials

### Animals and Housing

All procedures were approved by the Institutional Animal Care and Use Committee of Procter and Gamble Pet Care (Lewisburg, OH). A total of 12 black Labrador Retrievers (5 spayed females and 7 neutered males, 26.99 ± 0.63 kg; 4.9 ± 0.2 y; body condition score 3.0 on 5-point scoring scale with half points, adapted from [14]) were used in this study. All dogs resided at Procter and Gamble Pet Care (Lewisburg, OH) and were considered healthy based on a general health evaluation by a licensed veterinarian prior to study. Dogs were pair-housed in indoor runs (in the same building) with free access to water and indoor and outdoor runs. The indoor kennel (2.4 x 2.4 m) was maintained on a 12 h light (0600 h to 1800 h) and dark (1800 h to 0600 h) cycle, in addition to natural light. Indoor temperature was set at 22°C (range 18°C to 24°C) and humidity at 50% (range 40% to 70%) with 10 to 15 fresh air exchanges per hour. All indoor runs were equipped with raised canvas beds, toys and heated flooring. Outdoor runs (2.4 x 2.4 m) were equipped with toys and play yard equipment. All dogs received 40 min of supervised group exercise and socialization in a separate fenced yard daily (22.7 x 21.7 m).

### Study Design

This study was designed as a parallel, double-blind, cross-over with each dog receiving both dietary treatments, control (CON) and mannoheptulose (MH), in random order. The study took place January 2013 to July 2013 and included a ten week dietary adaptation period, two study periods (each 63 d), and a three week dietary washout between study periods. A summary of study measurements is presented in Table 1. Glucose and lipid kinetics were measured as part of this study but will be presented in a separate manuscript. Briefly, plasma glucose, glycerol, and palmitate turnover and oxidation were measured in the steady state using a stable isotope dilution methodology and indirect calorimetry. The scheduling of study events reflects the need to accommodate multiple technical staff personnel on a single day. Furthermore, the indirect calorimetry method only allowed for 4 dogs to be measured per day. Therefore, the 12 dogs were divided into 3 groups of 4 dogs with each diet represented on each day and staggered 1 d apart.

**Table 1:** Summary of study procedures.

Day of Study	Measurement/Procedure
Baseline ^1^	Continuous physical activity was monitored using an accelerometer
	Indirect calorimetry to determine energy expenditure and respiratory quotient
*Day 35* ^2^	*Glucose kinetics experiment*
Day 42	Indirect calorimetry to determine energy expenditure and respiratory quotient
Day 43	Dual x-ray absorptometry to determine body composition
Days 43–47	Continuous physical activity was monitored using an accelerometer
Day 49	Fasted muscle biopsy
Day 56	Post-prandial muscle biopsy
*Day 63* ^2^	*Lipid kinetics study*

^1^Baseline physical activity monitoring was conducted the week prior to initiation of each study period, indirect calorimetry measurements were made the 2 d and blood collection 1 d prior to the beginning of each study period

^2^ Glucose and lipid kinetics will be presented in a separate manuscript

### Diets and Feeding

Both diets, CON and MH, were made from identical ingredients and were similar in terms of nutrient content (Table 2). The MH diet was made by incorporating a water-soluble extract of flesh-only un-ripened fruit avocado (MH source) (Kemin Industries, Des Moines, IA) into the CON diet to deliver a dietary MH dose of approximately 400 mg/kg diet (extract described by [12, 15]). Four studies have administered MH orally to dogs. Labrador Retrievers received a dose of 2 mg/kg BW and plasma MH peaked 2 to 4 h after ingestion [16]. MH has also been given as an oral cocoa butter based supplement (8 mg/kg BW) to adult Beagles and plasma MH peaked 3 to 4 h after ingestion and an increase in post-prandial EE was observed [12]. Conversely, no changes in energy expenditure were noted in Labrador Retrievers fed a dietary dose of 2 mg/kg BW [12]. Animals in this study were fed an intermediate dietary dose of 400 mg/kg of diet (~6 mg/kg BW). This dose represents a cost effective dose that can be achieved in a commercial dry extruded pet food.

**Table 2:** Ingredient composition and proximate analysis of the control (CON) and mannoheptulose (MH) containing diets.

	CON	MH
**Ingredient inclusion, %**		
Chicken	17.7	17.7
Corn Meal	14.6	14.6
Chicken-By-Product Meal	14.4	14.4
Ground Whole Grain Sorghum	13.6	13.6
Corn Grits	11.1	11.1
Ground Whole Grain Barley	8.5	8.5
Fish Meal	5.4	5.4
Chicken Fat ^1^	4.0	4.0
Chicken Flavor	2.7	2.7
Beet Pulp	2.3	2.3
Mineral mix ^2^	1.9	1.9
Egg Product	0.9	0.9
Brewers Dried Yeast	0.9	0.9
Vitamin mix ^3^	0.5	0.5
Other ^4^	<0.5	<0.5
Avocado Extract	0.00	0.04
**Analyzed chemical profile (DM basis)**		
Dry matter, %	92.1	92.1
Crude protein, %	26.3	26.5
Crude fat, %	16.6	16.8
Crude fiber, %	2.2	2.3
Ash, %	6.5	6.6
ME ^5^, Kcal/g	3 705	3 708

^1^ Preserved with mixed tocopherols

^2^ Potassium chloride, calcium carbonate, sodium chloride, dicalcium phosphate, ferrous sulfate, zinc oxide, manganese sulfate, copper sulfate, manganous oxide, potassium iodide, cobalt carbonate

^3^ Vitamin E, choline chloride, ascorbic acid, vitamin A acetate, cross-linked beta carotene calcium pantothenate, biotin, thiamine mononitrate, vitamin B_12_, niacin, riboflavin, inositol, pyridoxine hydrochloride, vitamin D_3_, folic acid

^4^ Sodium hexametaphosphate, fructooligosaccharides, flax meal, dried chicken cartilage, DL-methionine, L-carnitine, rosemary extract

^5^ Metabolizable energy content (ME) was determined using the modified Atwater factors where fat, protein and carbohydrate provide 35.6, 14.7, 14.7 KJ/g (8.5, 3.5, and 3.5 kcal/g), respectively.

Animals were individually fed their daily ration in two meals (0700 h and 1300 h) and food intake was measured daily. Beginning 10 wk prior to study initiation and in between study periods (3 wk), dogs were fed the CON diet. A 10 wk dietary adaption period was selected partly because dogs included in the present study were involved in other research trials and fed diets of varying composition prior to the initiation of this study. Furthermore, given the duration of this cross-over study, a 10 wk adaption period was necessary to accommodate scheduling of study events and support staff. During the dietary wash-in period, energy intakes were adjusted to maintain body weight and a body condition score of 3.0. The day before study initiation, dogs were randomized to dietary treatment and energy intakes (1359 ± 13 Kcal/d) were fixed throughout the entire duration of the study (including in between treatment periods).

### Indirect Calorimetry

Respiratory gas exchange measurements were conducted via whole-body indirect calorimetry. The calorimetry chambers (76 cm × 53 cm × 61 cm, L × W × H) were made of clear plexiglass and fitted with a hinged access top door for providing food to the dog. Chambers were designed as open-flow circuits with room air pulled into the chambers at a rate of 22 to 26 L/min to maintain CO_2_ levels in the chamber between 0.4 and 0.8%. Exiting chamber air was dried by passing it through columns of Drierite™ and magnesium perchlorate before reaching the O_2_ and CO_2_ analyzers (Qubit Systems Inc., Kingston, ON).

Prior to study initiation, dogs were acclimated over an 8 wk period (1 to 8 h per week) to rest comfortably and calmly in the chamber with no excessive activity or movement. Dogs that did not acclimate were ineligible to participate in the study. Gas exchange measurements were conducted 2 d prior to the initiation of each study period (baseline) and on d 42 of each period. Prior to any measurements, the O_2_ and CO_2_ analyzers were calibrated with standard gases and dogs rested in the chamber for a minimum of 25 min to ensure adequate CO_2_ equilibration. Two fasting measurements were taken, after which dogs were fed their full daily ration of test diet as a single meal (time 0) and gas exchange measurements continued for 10 h. Animals were fed their full daily ration (opposed to their half ration) in order to maximize circulating MH concentrations and presumably its biological effects. Each chamber was sampled every 3 s over a 5 min period every 25 min. O_2_ and CO_2_ exchange and respiratory quotient data were logged in real time using data acquisition software (Qubit Systems Inc., Kingston, ON). Energy expenditure was calculated from O_2_ consumption and CO_2_ production (VO_2_ and VCO_2_) using the abbreviated Weir equation [17] and expressed on a per kg lean mass basis.

### Body Composition Analysis

On d 43 of each period, body composition analysis was completed using an X-Ray Bone Densitometer (QDR4500, Hologic Inc., Bedford MA). Dogs were fasted overnight (18 h since last meal) and sedated using Dexmedetomidine (Dexdomitor, Pfizer) at a dose of 0.02 mg/kg and Carprofen (Rimadyl, Pfizer) at a dose of 2 to 4 mg/kg administered i.m. Propofol (Propoflo, Abbott) at a dose of 5 to 7 mg/kg was administered i.v. for induction. Dogs were positioned on their sternum with the cranial aspect of ante brachium placed on the table to ensure the phalanges faced caudally. The hind limbs were extended with the tail placed straight and in between the hind limbs. A whole body scan was performed of the following regions: left arm, right arm, trunk, left leg, right leg and head. Scans were done in triplicate for each dog and the median value of the three scans was recorded. Following the scan, atipamezole (Antisedan®, Pfizer) was administered to each dog at a dose of 0.2 mg/kg. Dogs were placed in a heated cage until fully recovered and monitored for 1 wk for complications.

### Physical Activity Monitoring

Continuous physical activity measurements were made using the Actical accelerometer (Philips Respironics, Bend, OR). The Actical device (28mm × 27 mm × 10mm, ~17.5 g) was attached to the dog’s collar in the ventral position and all dogs were acclimated to wearing the device prior to study initiation. Raw activity values were converted to activity counts using ActiReader software (Philips Respironics, Bend, OR).

Baseline activity measurements were taken the week prior to study initiation and again the third week of the dietary washout period. Treatment measurements were taken on d 43 to 47 of each period (March 2013, period 1 and June 2013, period 2). The average activity per minute was calculated during dark (1800 h to 0600 h) and light (0600 h to 1800 h) time periods. The percent of active time was calculated by dividing the time when dogs were active (activities greater than or equal to 250 per minute) by the total time. Weekday (0600 h Wednesday through 0600 Saturday) and weekend (0600 h Saturday through 0600 h Monday) activity were analyzed separately, as there is less human interaction with the animals on weekends.

### Muscle Biopsy

Muscle biopsies were taken after an overnight fast (18 h since last meal) on d 49 of each treatment leg. On d 56 of both treatment legs, dogs were fed their full daily ration of test diet at their morning meal and muscle biopsies were taken 4 h post-feeding (contralateral leg to that of the fasted sample). This sampling time point coincides with peak MH concentrations in the plasma [13,14]. Animals were fed their full daily ration (opposed to their half ration) in order to maximize circulating MH concentrations and presumably its biological effects. Dogs were sedated using Dexmedetomidine (Dexdomitor, Pfizer) at a dose of 0.02 mg/kg and Carprofen (Rimadyl, Pfizer) at a dose of 2 to 4 mg/kg i.m. The hair over the incision point was clipped and the skin was prepared aseptically. A 1 to 2 cm incision was made with a #11 scalpel blade in the lateral aspect of the proximal thigh, and a Bergstrom needle (Surgipro, Inc. Shawnee, KS) was used to obtain 20–30 mg of muscle tissue from the vastus lateralis. The incision was closed using surgical skin staples (AutoSuture Appose ULC TM, Tyco) and atipamezole (Antisedan, Pfizer) was administered (0.2 mg/kg). Muscle samples were immediately frozen in liquid N_2_ and stored at –80°C for later immunoblot analysis.

### Western Blot

Muscle was homogenized in ice cold NP40 Cell Lysis Buffer (15:1 v:w) with 1 mmol/L PMSF (Invitrogen Corp., Camarillo CA) and protease inhibitor cocktail (Sigma, Oakville ON) and spun at 4°C for 5 min at 1500 x *g*. Supernatants were removed and protein concentration was determined by Bradford protein assay. Protein (25 μg) from muscle lysates was diluted in Laemmli sample buffer (Bio-Rad Laboratories, Mississauga ON) and loaded onto 7.5% Tris HCL pre-cast resolving gels (Bio-Rad Laboratories, Mississauga ON) and separated by SDS-PAGE. Gels were transferred onto nitrocellulose membranes (Bio-Rad Laboratories, Mississauga ON) (200 mA/tank). Membranes were blocked in 5% milk for 1 h at room temperature and washed in TBST for 10 min before being incubated in primary antibody overnight at 4°C. Primary antibodies (AMPK and pAMPK, Cell Signaling Technology Inc., Danvers, MA) were diluted (1:1000) in 5% BSA/TBST. Membranes were washed twice in TBST for 15 min and then incubated with anti-rabbit immunoglobulin–horseradish-peroxidase–linked secondary antibodies (1:2000 dilution in TBST) for ~1 h. The proteins of interest were visualized using enhanced chemiluminescence (GE Health Care Biosciences Corp., Piscataway, NJ) and captured and quantified using AlphaView Software for FluorChem Systems. All samples from individual dogs were loaded on to the same gel, in addition to a control sample. Data were normalized to the control sample and expressed as the ratio of phosphorylated to total protein content.

### Statistical Methods

A sample size of 12 was calculated from energy expenditure data reported by McKnight et al. (μ_1_ = 480 KJ/Kg^0.75^·d; μ_2_ = 447 KJ/Kg^0.75^·d; SD = 26 KJ/Kg^0.75^·d; α = 0.05; β = 0.8 [12]). All data were analyzed using SAS version 9.2 (SAS Institute, Cary, NC) and are expressed as means and pooled SEM. Mixed effects models were fitted using the PROC MIXED procedure of SAS assuming fixed period and diet effects and dogs as random variables. Denominator degrees of freedom were calculated using the Kenward-Rogers approximation. Repeated measures within period on dog were analyzed using the autoregressive order 1 covariance structure. Multiple comparisons were made using the Tukey-Kramer method. Interactions between fixed effects were tested but only discussed if significant. For calorimetry data, fasting, 0 to 10 h post-prandial and 3 to 5 h post-prandial data were analyzed separately and time was considered a fixed effect. MH concentrations have been shown to peak in plasma between 3 to 5 h after feeding and the effects of MH on metabolism are transient. Therefore, the post-prandial time period of 3 to 5 h was selected to examine maximal MH effects, and in addition, to provide a basis of comparison for muscle protein analysis. Results were considered statistically significant if p < 0.05.

## Results

### Body Weight and Composition

All dogs maintained good general health, with no instances of diarrhea or poor fecal quality, throughout the entire study. Food intake was fixed in this study based on the dogs historical energy intake records and was complete (no remaining orts) for all dogs throughout the entire study.

**Fig 1:**
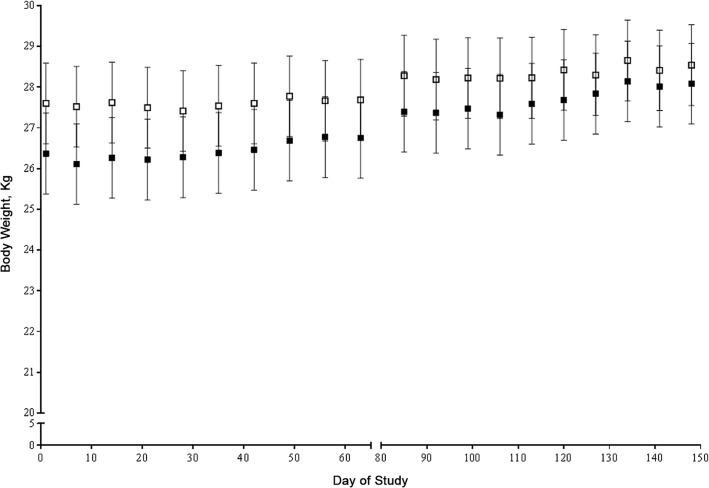
Weekly body weights (kg). Dogs were randomized to dietary treatment order, a MH containing diet (MH, 400 mg/kg) followed by control diet (CON) (■) (n = 6) or CON followed by MH (□) (n = 6), with a 3 wk dietary washout between periods.

Body weight significantly increased throughout the study (p _time_ < 0.01) irrespective of diet (p _diet_ = 0.27) (Fig 1) and there was no interaction between diet and time (p = 0.35). Body composition is presented in Table 3. Diet did not significantly affect body weight, fat or lean mass. However, dogs fed MH had a significantly lower ratio of fat to lean mass. There was a significant affect of period for body weight, fat mass, and percent fat mass, which were all higher in the second period. Percent lean mass was lower in the second period (Table 3), but absolute lean mass did not differ between treatments or periods. These findings are not surprising given that dogs gained weight throughout the study.

**Table 3:** Body composition (d 43) measured by dual-energy X-ray absorptiometry of adult Labrador Retrievers fed either a control diet (CON, no mannoheptulose) or a mannoheptulose containing diet (MH, 400 mg/kg) (a total of 12 dogs in a complete cross-over design).

	Diet		Period		SEM	*P*-value	
	MH	CON	1	2		Diet	Period
Body weight, kg	27.5	28.2	27.1	28.6	0.65	0.11	< 0.05
Lean mass, kg	21.3	21.4	21.2	21.5	0.56	0.25	0.13
Fat mass, kg	5.23	5.45	4.87	5.80	0.25	0.30	< 0.05
Lean mass, %	77.4	76.8	78.3	75.9	0.87	0.23	< 0.05
Fat mass, %	17.0	19.0	18.0	20.6	1.27	0.25	< 0.05
Fat: lean, %	24.7	27.7	23.1	29.3	1.7	0.03	< 0.05

### Energy Expenditure and Respiratory Quotient

There were no differences in baseline measures of fasting or post-prandial EE or respiratory quotient (RQ) between dietary groups (S1 Table). Resting energy expenditure (REE) was not affected by diet, but was higher (p = 0.03) in the first period than the second period (179 vs. 160 kJ/(kg _lean mass_ · d), respectively) (Table 4). Post-prandial (0 to 10 h) EE was not significantly different between diets (Table 4). From 3 to 5 h post-prandial, EE tended (p = 0.08) to be lower in dogs fed MH than those fed CON. Diet did not affect fasting or post-prandial (0 to 10 h) RQ; however, during 3 to 5 h post-prandial, RQ was significantly lower in dogs fed MH compared to dogs fed CON.

**Table 4:** Resting and post-prandial energy expenditure (EE) and respiratory quotient (RQ) (d 42) in adult Labrador Retrievers fed either a control (CON, no mannoheptulose) or mannoheptulose containing diet (MH, 400 mg/kg) (a total of 12 dogs in a complete cross-over design).

	Diet		Period			*P* value		
	MH	CON	1	2	SEM	Diet	Period	Time
**EE, Kcal/(kg**_**lean mass**_ **· d)**								
Resting	39.6	41.5	42.9	38.3	1.9	0.31	0.03	-
Post-prandial (0–10 h)	62.9	64.9	66.8	65.7	1.7	0.16	0.31	< 0.05
Post-prandial (3–5 h)	64.6	67.9	64.6	63.2	1.7	0.08	0.55	0.01
**RQ**								
Fasting	0.77	0.76	0.76	0.76	0.01	0.44	0.47	-
Post-prandial (0–10 h)	0.87	0.87	0.87	0.86	0.01	0.31	0.20	< 0.05
Post-prandial (3–5 h)	0.87	0.88	0.88	0.87	0.01	0.02	0.15	< 0.05

### Physical Activity

For all baseline and period activity measurements, dogs were more active in light than dark time periods (S2 Table, Table 5).

**Table 5:** Spontaneous physical activity counts as measured using an accelerometer in adult Labrador Retrievers fed either a control (CON, no mannoheptulose) or mannoheptulose containing diet (MH, 400 mg/kg) (a total of 12 dogs in a complete cross-over design).

	Diet		Period		SEM	*P* value	
	MH	CON	1	2		Diet	Period
**Weekday** ^**1**^							
Dark, activity/min ^2^	71	70	100	40	9	0.92	< 0.05
Light, activity/min ^3^	251	272	258	265	25	0.16	0.60
Dark, % ^4^	6.0	6.0	8.0	4.0	1.0	0.88	< 0.05
Light, %	19	20	19	19	2.0	0.37	0.66
**Weekend** ^**5**^							
Dark, activity/min	66	63	87	42	7	0.80	< 0.05
Light, activity/min	223	258	233	248	16	< 0.05	0.14
Dark, %	6.0	6.0	7.0	4.0	1.0	0.87	< 0.05
Light, %	17	19	16	19	1.0	0.14	0.01

^1^ Weekday = measurements taken 0600 h Wednesday through 0600 h Saturday

^2^ Dark = measurements taken from 1800 h – 0600 h

^3^ Light = measurement taken from 0600 h – 1800 h

^4^ Percent of active time = activities greater than or equal to 250 per minute divided by the total time

^5^ Weekend = measurements taken 0600 h Saturday through 0600 h Monday

Weekday and weekend baseline activity per minute was not different between diets prior to study initiation or during the dietary washout period (S2 Table). Similarly, there was also no effect of diet on percent of time activity during baseline measurements (Table 5).

Period was significant for weekday and weekend activity per minute and percent active time in the dark time period (Table 5). For both weekday and weekend, activity measures were higher in the first period, compared to the second period. There was no statistically significant effect of diet on weekday activity per minute (light or dark). However, daytime activity per minute was significantly lower in dogs fed MH than those fed control (Table 5). There was no significant effect of diet on percent of time active in dark or light periods (Table 5).

### Skeletal Muscle Protein Abundance

**Fig 2:**
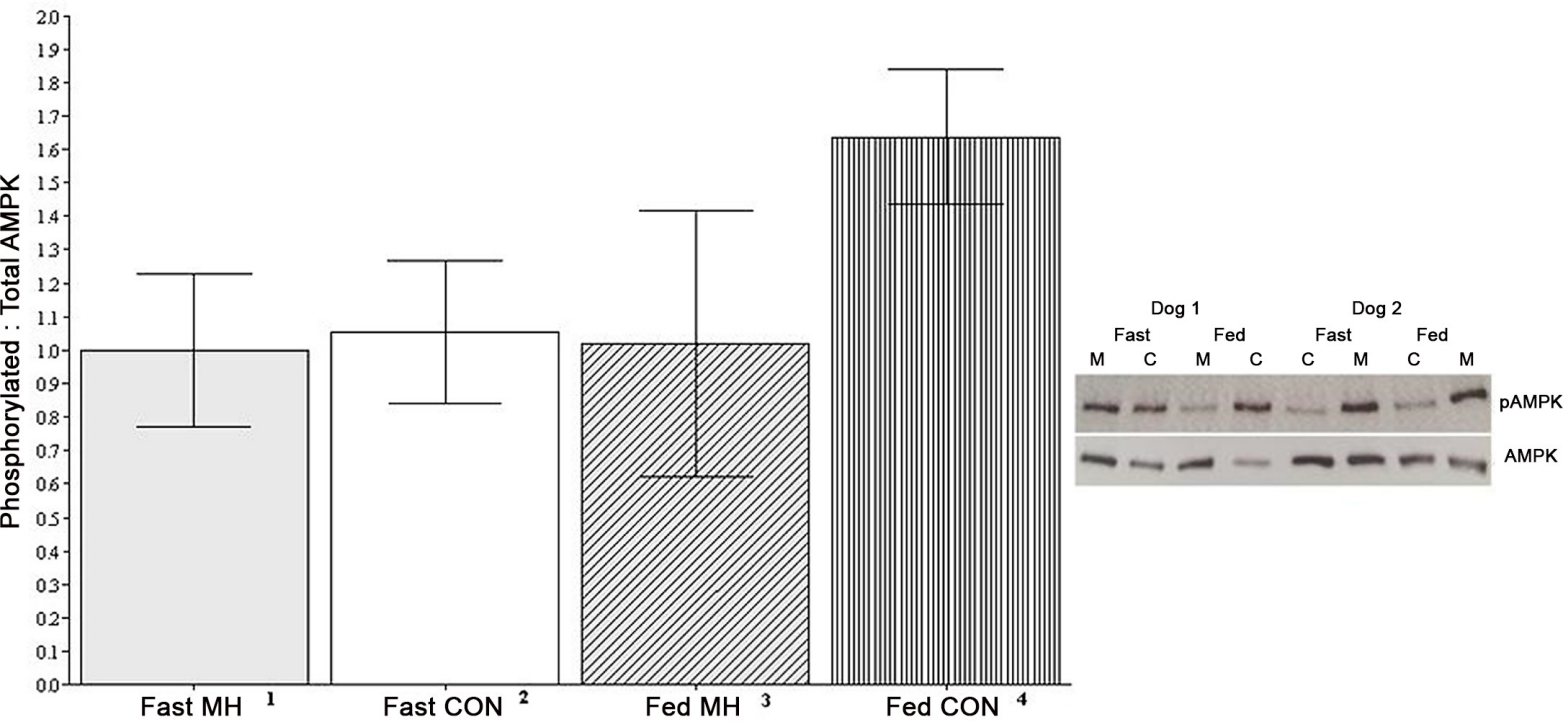
The ratio of phosphorylated to total AMPK (pAMPK/total) protein abundance in vastus lateralis muscle of adult Labrador Retrievers. **Fasting muscle samples were taken prior to the dog’s morning meal (18 h since last meal). Fed muscle samples were taken 4 h after the dogs consumed their full daily ration of test diet, either control or mannoheptulose (400 mg/kg).** Data are means with pooled standard errors. ^1^ N = 9; ^2^ N = 9; ^3^ N = 7; ^4^ N = 10

An inadequate amount of muscle tissue (< 20 mg) was harvested to complete the necessary analyses in some animals and as such, sample sizes were reduced across all treatments. AMPK was expressed as the ratio of phosphorylated to total AMPK protein content (Fig 2). There was no significant effect of diet or period on fasting (1.0, N = 9 MH; 1.1, N = 9 CON; SEM 0.2, p = 0.72,) or post-prandial (1.0, N = 7, MH; 1.6, N = 10 CON; SEM 0.3, p = 0.24) AMPK.

## Discussion

Dietary MH (400 mg/kg of diet; 6 mg/kg BW), delivered as an extract of the flesh of un-ripened avocados significantly decreased post-prandial RQ andvoluntary physical activity in adult Labrador Retrievers. These MH induced changes were not associated with alterations in EE or the content and phosphorylation of AMPK in skeletal muscle. Interestingly, decreased physical activity associated with MH feeding and led to a reduction in body fat to lean mass, without altering body weight (despite equivalent energy intakes). Energy expenditure as measured by indirect calorimetry includes obligatory and facultative components of REE, adaptive thermogenesis and a small amount of voluntary activity. It does not include the energy expended during voluntary physical activity. REE represents the major component of total EE (~70%) and is largely predicted by fat free mass [18]. As lean mass was not affected by diet in this study, REE was not expected to be affected by diet. The REE values observed in this study are similar to those reported in Beagles [12, 19] and Labrador Retrievers [12, 20]. Adaptive thermogenesis includes the energy produced in response to environmental temperature, behaviour, and diet and accounts for a relatively small proportion of total EE (~10%). MH had no significant effect on adaptive thermogenesis in the present study. This finding is in contrast to McKnight et al. [12] who noted an increase in post-prandial EE in Beagles fed a MH supplement (8 mg/kg). There are several key differences between these two studies. For example, the current study used Labrador Retrievers, whereas, McKnight et al. [12] utilized Beagles. Metabolic differences between the two breeds have been reported [21]. Furthermore, the diets used in the two studies were considerably different with respect to dietary macronutrient composition and ingredients. Using the present EE results, a sample size of 44 dogs would be required.

As MH has been shown to inhibit hexokinases, the reduced flux through glycolysis was expected to limit glucose as a substrate of energy production and consequently increase fat utilization. In agreement, post-prandial RQ was transiently reduced in dogs fed MH, suggesting a higher proportion of fat relative to carbohydrate oxidation. However, RQ does not indicate net carbohydrate or fat oxidation, nor does it account for protein oxidation. Therefore, more sensitive measures of substrate oxidation are warranted. Inhibition of glycolysis may lead to a decrease in intracellular ATP. Lowering the ATP to ADP ratio would activate AMPK. Once activated, AMPK increases ATP production by potently promoting fat oxidation. Indeed, glucose deprivation, as imposed by another glycolytic inhibitor, 2-deoxyglucose, has been shown to activate AMPK [22, 23]. In this study, MH had no effect on the ratio of phosphorylated to total AMPK protein content (used as a marker of AMPK activation) in skeletal muscle. It is important to note that an inadequate amount of muscle tissue was collected to complete the required analysis in all treatments, thereby reducing statistical power and the opportunity to detect significant differences. Glucose utilization in skeletal muscle is mediated by hexokinase II isoform, which has a low *K*_*m*_ for glucose compared to that of hexokinase IV found in liver, pancreas and brain. As glucose phosphorylation in skeletal muscle is saturated at low glucose concentrations, AMPK may not be as sensitive to changes in glucose supply as it would be in tissues that express glucokinase. In addition, MH was delivered in a mixed meal. Therefore, it is also likely that effectiveness of MH as competitive inhibitor of hexokinase II was overcome due to the abundance of dietary derived carbohydrate (glucose). A dose response study is necessary to better understand the role of dietary MH as a glycolytic inhibitor in skeletal muscle and other tissues, particularly adipose tissue, given the observed reduction in fat to lean body mass in the present study.

The final component of EE, physical activity, can account for 20–30% of EE. Dogs in this study exhibited diurnal patterns of activity; specifically dogs were more active during the light than dark periods (irrespective of diet). This finding agrees with the activity patterns displayed by laboratory Beagles [24]. In contrast, feral dogs have been shown to be more active in dark periods than light periods [25]. Differences in physical activity patterns between laboratory housed dogs and feral dogs suggest that dogs are able to adapt activity patterns to their environmental conditions. In the dark time period, dogs were more active in the first study period (March 2013) than the second period (June 2013). This finding may be attributed to differences in light exposure and intensity between study periods. Specifically, exposure to natural light was reduced in the first period compared to the second period due to the time of year. The reduced exposure to natural light would have imposed an abrupt transition between light and dark periods, as artificial lighting was maintained on a 12 h cycle. Previous research in Beagles has demonstrated that dogs exhibit anticipatory activity prior to the lights coming on in an indoor housing arrangement [24]. Study period did not affect daytime activity patterns. This finding is in agreement with Siwak et al. [24] who found daytime activity patterns in laboratory Beagles to be largely dictated by standardized daily routine that dogs experienced in their housing facility. In contrast, MH decreased daytime physical activity on the weekend when there is reduced human-animal contact. It is important to note that activity, as measured in this study, represents the mean activity count (locomotion) over the entire light (06:00–18:00) and dark periods (18:00–06:00). It is unclear how MH affects discrete activity bouts and conversely rest periods, but this finding is interesting. Furthermore, locomotion encompasses complex behaviours (i.e., spontaneous activity, exploration and exercise) that are elicited by a wide range of internal and external stimuli. A more critical analysis of physical activity and related behaviours is necessary to fully understand the effects of MH.

With prolonged reductions in physical activity, as imposed by daily MH feeding, one may expect weight gain. However, MH decreased the ratio of body fat to lean mass, despite all dogs having equivalent energy intakes. To understand the impact of MH on EE better, other measurement techniques, including the doubly labelled water technique, should be considered. The doubly labelled water technique would enable average estimates of EE over variable time periods in the dog’s regular environment. Furthermore, other sources of energy loss including fecal and urinary losses, which were not measured in the current study, should be examined.

Irrespective of MH, study period had a significant impact on several study outcomes, including body weight. Dogs in this study were fed to maintenance weight based on historical energy intake records yet still gained weight. Basing energy intakes on historical energy intake records is a common practice in companion animal nutrition, largely due to the impracticality of using more sensitive measures such as indirect calorimetry. However, the accuracy of energy intake records is debatable, especially for studies of long duration such as the present one. The primary reason for fixing energy intakes in this cross-over designed study (opposed to adjusting intakes weekly to maintain body weight) was to ensure individual dogs received an equivalent amount of MH in each study period. The body weight gain observed in this 6 month study was modest, approximately 3% of the animal’s body weight (equivalent to a 2 kg increase in 70 kg adult). As lean mass was not different between study periods, the gain in body mass was considered fat deposition. Fat deposition was likely influenced by reduced physical activity observed in the second study period. Seasonal changes, including differences daylight exposure and climate, between period 1 (winter) and period 2 (spring) may have influenced animal activity patterns and in turn body composition. Seasonal changes in fat deposition have been noted in ruminants [26–28] and recently cats [29]. However, information related to seasonal changes in physical activity and fat deposition in dogs is lacking.

## Conclusions

A modest but statistically significant transient reduction in post-prandial RQ was observed in dogs fed MH. The biological significance of the decrease in RQ is uncertain. It may be reflective of increased fat oxidation, as a reduction in fat to lean body mass with MH feeding was observed (despite equivalent energy intakes). However, changes in body composition with daily MH feeding (~6 mg/kg BW) were likely influenced by decreased voluntary physical activity, particularly on the weekend when there is reduced human animal contact. As physical activity encompasses a broad range of complex behaviours, a more critical analysis of physical activity (and related behaviours) is warranted. These results suggest that MH affects energy balance of adult dogs, but that these effects are not dose dependent and not due to physical activity.

## Supporting Information

S1 Data (XLS)

S1 Table Baseline^1^ measures of resting and post-prandial energy expenditure (EE) and respiratory quotient (RQ) (d 42) in adult Labrador Retrievers fed either a control (CON, no mannoheptulose) or mannoheptulose containing diet (MH, 4 mg/kg BW) (a total of 12 dogs in a complete cross-over design). (DOCX)

S2 Table Baseline^1^ measures of spontaneous physical activity counts as measured using an accelerometer in adult Labrador Retrievers fed either a control (CON, no mannoheptulose) or mannoheptulose containing diet (MH, 4 mg/kg) (a total of 12 dogs in a complete cross-over design). (DOCX)
