# Supplementary material for: Dietary Mannoheptulose Does Not Significantly Alter Daily Energy Expenditure in Adult Labrador Retrievers
Source: PLoS One. 2015 Dec 11;10(12):e0143324. doi: 10.1371/journal.pone.0143324 (PMC4684352; doi:10.1371/journal.pone.0143324)
Supplement: S1 Table — (DOCX) [file pone.0143324.s002.docx]

**S1 Table. Baseline^1^ measures of resting and post-prandial energy expenditure (EE) and respiratory quotient (RQ) (d 42) in adult Labrador Retrievers fed either a control (CON, no mannoheptulose) or mannoheptulose containing diet (MH, 4 mg/kg BW) (a total of 12 dogs in a complete cross-over design).**

|  | |  | CON | MH | SEM | P |
| --- | --- | --- | --- | --- | --- | --- |
| **Baseline Measurements (wash in)** | | |  |  |  |  |
| EE, Kcal/(kg^0.75^·d) | Resting | | 78.3 | 88.7 | 5.3 | 0.20 |
|  | Post-prandial (3 – 5 h) | | 121.0 | 125.5 | 8.6 | 0.83 |
| RQ | Resting | | 0.78 | 0.78 | 0.01 | 0.67 |
|  | Post-prandial (3 – 5 h) | | 0.87 | 0.86 | 0.01 | 0.46 |
| **Baseline Measurements (wash out)** | | |  |  |  |  |
| EE, Kcal/(kg^0.75^·d) | Resting | | 80.0 | 78.8 | 3.7 | 0.46 |
|  | Post-prandial (3 – 5 h) | | 127.7 | 123.4 | 3.3 | 0.57 |
| RQ | Resting | | 0.75 | 0.75 | 0.01 | 0.96 |
|  | Post-prandial (3 – 5 h) | | 0.86 | 0.86 | 0.01 | 0.57 |

^1^ Baseline measurements were taken 2 d prior to the initiation of each study period
