# Supplementary material for: Dietary Mannoheptulose Does Not Significantly Alter Daily Energy Expenditure in Adult Labrador Retrievers
Source: PLoS One. 2015 Dec 11;10(12):e0143324. doi: 10.1371/journal.pone.0143324 (PMC4684352; doi:10.1371/journal.pone.0143324)
Supplement: S2 Table — (DOCX) [file pone.0143324.s003.docx]

**S2 Table. Baseline^1^ measures of spontaneous physical activity counts as measured using an accelerometer in adult Labrador Retrievers fed either a control (CON, no mannoheptulose) or mannoheptulose containing diet (MH, 4 mg/kg) (a total of 12 dogs in a complete cross-over design).**

|  |  | **MH** | **CON** | **SEM** | **P** |
| --- | --- | --- | --- | --- | --- |
| **Baseline Measurements (wash in)** | |  |  |  |  |
| Weekday | Dark, activity/min | 30 | 40 | 6 | 0.20 |
|  | Light, activity/min | 312 | 353 | 38 | 0.46 |
|  | Dark, % | 3 | 4 | 1 | 0.36 |
|  | Light, % | 23 | 24 | 2 | 0.79 |
| Weekend | Dark, activity/min | 37 | 52 | 7 | 0.17 |
|  | Light, activity/min | 292 | 308 | 32 | 0.74 |
|  | Dark, % | 4 | 5 | 1 | 0.31 |
|  | Light, % | 22 | 22 | 3 | 0.94 |
| **Baseline Measurements (wash out)** | |  |  |  |  |
| Weekday | Dark, activity/min | 40 | 34 | 6 | 0.54 |
|  | Light, activity/min | 330 | 330 | 38 | 1.0 |
|  | Dark, % | 4 | 4 | 1 | 0.51 |
|  | Light, % | 22 | 23 | 1 | 0.86 |
| Weekend | Dark, activity/min | 38 | 35 | 6 | 0.74 |
|  | Light, activity/min | 384 | 389 | 44 | 0.90 |
|  | Dark, % | 4 | 4 | 1 | 0.52 |
|  | Light, % | 23 | 26 | 2 | 0.42 |

^1^ Baseline measurements were taken the week preceding the initiation of each study period; Weekday = measurements taken 0600 h Wednesday through 0600 h Saturday; Dark = measurements taken from 1800 h – 0600 h; Light = measurement taken from 0600 h – 1800 h; Percent of active time = activities greater than or equal to 250 per minute divided by the total time; Weekend = measurements taken 0600 h Saturday through 0600 h Monday

**Supporting information:** Raw data is included in Supporting Information (PlosOne Data.xls)
